# BMP-6 inhibits growth of mature human B cells; induction of Smad phosphorylation and upregulation of Id1

**DOI:** 10.1186/1471-2172-6-9

**Published:** 2005-05-09

**Authors:** Christian Kersten, Einar A Sivertsen, Marit E Hystad, Lise Forfang, Erlend B Smeland, June H Myklebust

**Affiliations:** 1 Department of Immunology, Institute for Cancer Research, The Norwegian Radium Hospital, Montebello, 0310 Oslo, Norway; 2 Faculty Division The Norwegian Radium Hospital, University of Oslo, Norway

## Abstract

**Background:**

Bone morphogenetic proteins (BMPs) belong to the TGF-β superfamily and are secreted proteins with pleiotropic roles in many different cell types. A potential role of BMP-6 in the immune system has been implied by various studies of malignant and rheumatoid diseases. In the present study, we explored the role of BMP-6 in normal human peripheral blood B cells.

**Results:**

The B cells were found to express BMP type I and type II receptors and BMP-6 rapidly induced phosphorylation of Smad1/5/8. Furthermore, Smad-phosphorylation was followed by upregulation of *Id1 *mRNA and Id1 protein, whereas Id2 and Id3 expression was not affected. Furthermore, we found that BMP-6 had an antiproliferative effect both in naïve (CD19^+^CD27^-^) and memory B cells (CD19^+^CD27^+^) stimulated with anti-IgM alone or the combined action of anti-IgM and CD40L. Additionally, BMP-6 induced cell death in activated memory B cells. Importantly, the antiproliferative effect of BMP-6 in B-cells was completely neutralized by the natural antagonist, noggin. Furthermore, B cells were demonstrated to upregulate *BMP-6 *mRNA upon stimulation with anti-IgM.

**Conclusion:**

In mature human B cells, BMP-6 inhibited cell growth, and rapidly induced phosphorylation of Smad1/5/8 followed by an upregulation of Id1.

## Background

Members of the transforming growth factor β (TGF-β) superfamily play central roles in controlling cellular proliferation, differentiation, migration and apoptosis [1]. These cytokines can be divided into three subgroups: TGF-β, the activins/inhibins, and the bone morphogenetic proteins (BMPs), of which the latter constitute the largest family. BMPs are 30–38 kDa hetero- or homodimeric proteins originally identified by their ability to induce ectopic cartilage and bone formation [2,3]. Several studies have demonstrated an essential role of these proteins during embryogenesis, and more recently, also in adult tissues [1]. TGF-β has been intensively studied in normal and malignant haematopoietic cells and is one of the most potent endogenous negative regulators known to date. [4]. In contrast, the effect of BMPs in the immune system has not been widely investigated. In that respect, BMP- 2, -4 and -7 have been found to control differentiation of hematopoietic stem cells [5] and early T cell development [6,7]. BMP-6 has been reported to reduce the number of cobblestone-area-forming cells of normal human haematopoietic cells [8]. Furthermore, BMP-2, -4, 6 and -7 had an antiproliferative and a proapoptotic effect on multiple myeloma cells [9-11]. In addition, by gene expression profiling, BMP-6 significantly increased the predictive value for a multi-gene signature test and was associated with a poor outcome in diffuse large B cell lymphomas (DLBCL) [12].

BMP-6, like the other BMP members, signals through ligation and heterodimerzation of BMP type I [activin-like-kinase (ALK)] and type II serine-threonine kinase receptors, which subsequently propagates the signal downstream by phosphorylating Smad proteins. BMP-6 can signal through the ligation of the type I receptors Act-RIA, BMP-RIA, and BMP-RIB and the type II receptors BMP-RII, Act-RIIA and Act-RIIB, which lead to the phosphorylation of the receptor Smads (Smad-1, Smad-5, and Smad-8). The R- Smads then form complexes with the co-Smad (Smad4) and are translocated into the nucleus where they exert gene regulation [1,13].

Given the reported role of BMP-6 in B-cell malignancies and haematopoietic progenitor cells, we wanted to explore its potential role in normal human B cells. We studied the effects of BMP-6 on proliferation and apoptosis on resting and stimulated B cells. Furthermore, the expression of BMP receptors and BMP-6 induced activation of the Smad signalling pathway with subsequent regulation of the target genes *Id1–Id4*, were resolved. Finally, we investigated whether B cells also were capable of producing BMP-6.

## Results

### BMP-6 inhibits anti-IgM induced proliferation of human B cells

**Figure 1:**
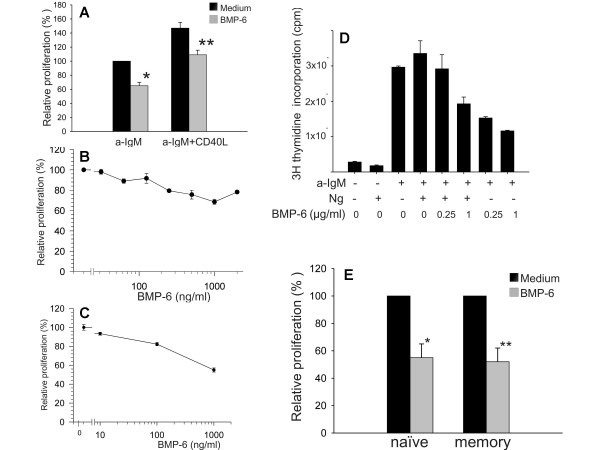
**BMP-6 inhibits proliferation of human B cells**. **A) **CD19^+ ^B cells were isolated from peripheral blood and stimulated in triplicates with anti-IgM (37.5 μg/ml) or anti-IgM and CD40 ligand (CD40L; 10 ng/ml) in the presence or absence of BMP-6 for 72 hours. DNA synthesis was measured by [3H]-thymidine incorporation for the last 18 hours. Data are given as relative proliferation obtained by normalizing the mean counts per minute (cpm) for each stimulation to the mean cpm obtained for anti-IgM stimulated cells ± SEM. (mean cpm = 25 352 for anti-IgM stimulated cells, *p ≤ 0.0002 (n = 8), **p ≤ 0.023 (n = 6). **B) **Dose dependent inhibition of BMP-6 of anti-IgM induced DNA-synthesis of CD19^+ ^B cells (relative proliferation ± SEM, n = 3) and **C) **the Burkitt lymphoma cell line Ramos (relative proliferation ± SEM, n = 3). Ramos cells were cultured for 72 hours and [3H]-thymidine were added for the last 4 hours. **D) **Noggin (5 μg/ml) and BMP-6 (0,25 or 1 μg/ml) were preincubated for 1 h at 37°C and then added to the CD19^+ ^B cells in the presence of anti-IgM(37.5 μg/ml). Cells were cultured for 72 h and DNA synthesis was measured by ^3^H-thymidine incorporation. One representative of three separate experiments is shown (mean cpm ± SD of triplicates). **E) **Highly purified CD19^+^CD27^- ^or CD19^+^CD27^+ ^cells were obtained by cell sorting of CD19^+ ^cells and stimulated with anti-IgM in the presence or absence of BMP-6, as indicated for 72 hours. DNA synthesis was measuredby [3H]-thymidine incorporation. Data are given as relative proliferation obtained by normalizing the mean cpm for each stimulation to the mean cpm obtained for anti-IgM stimulated cells (mean cpm = 18 221 for CD19^+^CD27^- ^naïve B cells, mean cpm = 8 930 for CD19^+^CD27^+ ^memory B cells, n = 5; * p ≤ 0,004, **p ≤ 0.001).

The effects of BMP-6 on normal and neoplastic hematopoietic cells prompted us to investigate the effects of BMP-6 on normal human B cells. All experiments in this study were performed under serum-free conditions as FCS has been shown to interfere with BMP-signalling [14](own observations). To study the effect of BMP-6 on proliferation, B-cells from healthy volunteers were stimulated with anti-IgM and/or CD40L in the presence or absence of BMP-6 for three days. We found that BMP-6 led to a 35% mean reduction of anti-IgM- induced DNA synthesis (n = 8; p ≤ 0.0002, Figure 1A). Similar results were obtained for B cells treated with anti-IgM and CD40L (26% mean reduction, n = 6; p ≤ 0.023). The BMP-6-induced inhibition of proliferation was dose-dependent in both peripheral B cells (Figure 1B) and the Burkitt lymphoma cell line Ramos (40% reduction of DNA synthesis, Figure 1C). The BMP-6 effects could be reversed by addition of the extracellular inhibitor Noggin (Figure 1D). Similarly, a combination of the soluble BMP receptors BMP-RIB-Fc and BMP-RII-Fc also neutralized the effects of BMP-6 (data not shown). Next, we wanted to test whether BMP-6 had different effect on naïve and memory B cells. Naïve (CD19^+^CD27^-^) and memory (CD19^+^CD27^+^) B cells were isolated from peripheral blood by cell sorting of immunobead-isolated CD19^+ ^B cells [15], and tested for their capacity to proliferate in the presence of BMP-6. However, BMP-6 inhibited anti-IgM induced DNA synthesis in the two subpopulations to a similar extent, with a mean reduction of DNA-synthesis of 45% (n = 5; p ≤ 0,004) for naïve B cells and 48% (n = 5; p ≤ 0,001) for memory B cells (Figure 1E).

### BMP-6 induces cell death in human memory B cells and Ramos cells

**Figure 2:**
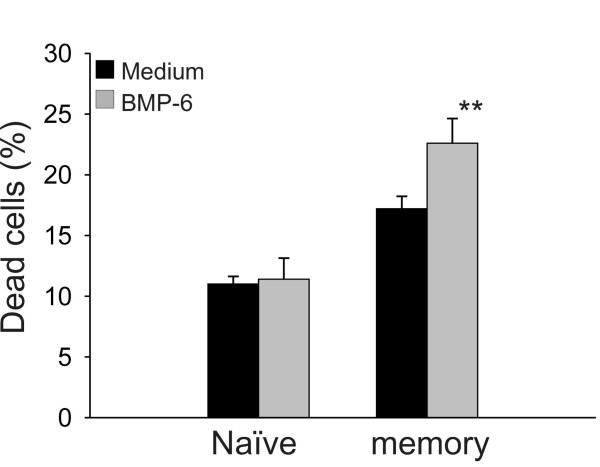
**BMP-6 induces cell death in B cells**. CD19^+^CD27^- ^naïve B cells or CD19^+^CD27^+ ^memory B cells were cultured for 48 h with BMP-6 (1 μg/ml) with or without anti-IgM (37.5 μg/ml). Cell death (PI^+ ^cells) was then measured by flow cytometry analysis. Data are shown as mean percentage PI^+ ^cells from five independent donors (± SEM; p ≤ 0,003).

**Figure 3:**
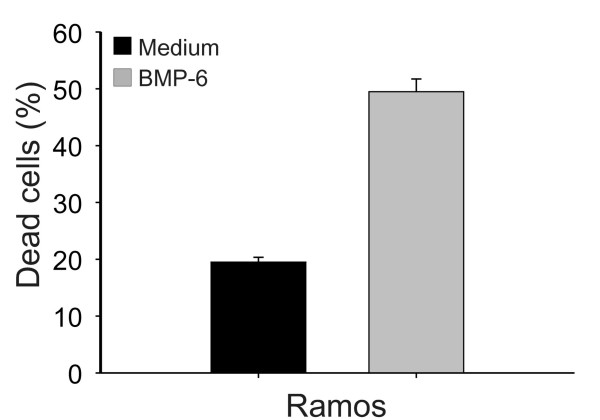
Ramos cells were cultured in the presence or absense of BMP-6 (1 μg/ml) for 48 h before analysis of cell death by PI staining. Data are shown as mean percentage PI^+ ^cells (± SEM, n = 3, p ≤ 0,001).

Next, we wanted to establish whether BMP-6 also could affect the viability of normal B cells. Cell viability was determined by propidium iodide (PI) staining after culture with or without BMP-6 for 48 hours. Interestingly, BMP-6 showed a small, but reproducible mean increase of cell death from 17 to 23% (n = 5; p ≤ 0,003) in anti-IgM stimulated CD27^+ ^memory B cells. Furthermore, Ramos cells showed a mean increase in cell death from 20 to 50% (n = 3, p < 0,001, figure 3) after BMP-6 treatment. In contrast, cell death of total CD19^+ ^cells (n = 6; p ≤ 0,32; data not shown) or CD27^-^IgG^- ^naïve B cells was not significantly affected (n = 5; p ≤ 0,65, figure 2).

### Human B cells express BMP-6 receptors

**Figure 4:**
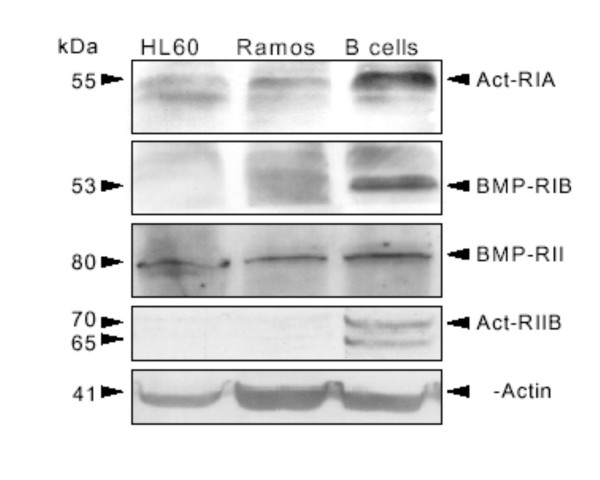
**CD19^+ ^B cells express BMP type I and type II receptors**. Total cell lysates were prepared from the myeloid cell line HL60, the cell line Ramos or CD19^+ ^B cells isolated from peripheral blood and analyzed by western blot for expression of BMP type I and type II receptors. One representative experiment of four is shown.

Detailed knowledge regarding expression of different BMP receptors in B cells is currently not available. To further elucidate the role of BMPs in human B cells, we performed western blot analysis for type I and type II BMP receptors. This analysis revealed that the type I receptors Act-RIA, BMP-RIB and the type II receptors BMP-RII and Act-RIIb are expressed on resting human B-cells (Figure 4). Ramos cells expressed the type I receptors Act-RIA, weakly BMP-RIB and the type II receptor BMP-RII, but more weakly than normal B cells (Figure 4). HL60 cells were used for comparison and weakly expressed Act-RIA and BMP-RII.

Taken together, these data show that normal human B cells and Ramos cells express a set of BMP receptors, previously shown to bind BMP-6 [16].

### BMP-6 induces phosphorylation of Smad1/5/8

**Figure 5:**
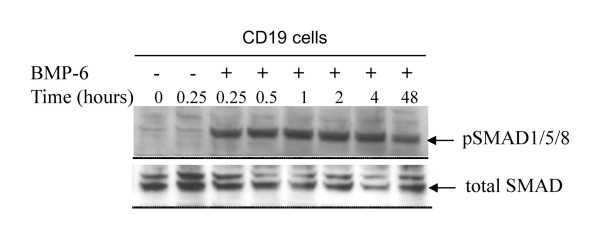
**BMP-6-induced phosphorylation of Smad1/5/8 in CD19^+ ^B cells and Ramos, but not in HL60 cells**. CD19^+ ^B cells, or the cell lines Ramos or HL60

**Figure 6:**
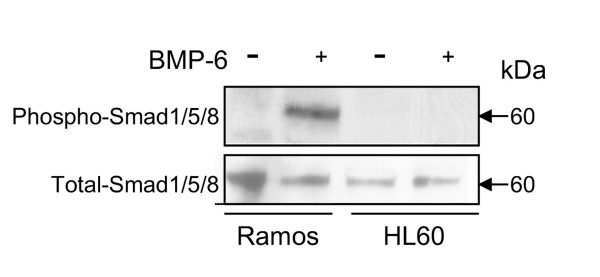
were cultured in X-vivo 15 over night before treatment with BMP-6 for 30 minutes, or for the indicated time points before total cell lysates were prepared. The amount of phosphorylated Smad 1/5/8 was determined by western-blot analysis. The membranes were reprobed for Smad1. One representative experiment of three is shown.

Upon ligand binding, the type II receptor transphosphorylates and activates the type I receptor. Type I receptors can signal via several pathways. We examined the effect of BMP-6 on Smad phosphorylation, as the activation of Smad is considered to be a major signalling pathway for BMPs [17]. B cells were cultured in serum-free media over night and then treated with BMP-6 for various time points. Total protein lysates were prepared, and the amounts of the phosphorylated forms of Smad1/5/8 were determined by western blot analysis. Interestingly, treatment with 500 ng/ml BMP-6 induced phosphorylation of Smad. The BMP-6 induced phosphorylation was high at the earliest time point tested (15 minutes), and remained high for at least 48 hours (Figure 5). A similar phosphorylation was observed in Ramos cells, but not in HL60 cells (Figure 6). Furthermore, we also tested whether other known downstream signalling pathways of BMP-6 could be triggered by BMP-6 in human B cells. However, we did not observe any significant changes in the level of phospho-STAT3 or phospho-p38 upon BMP-6 treatment of B cells (data not shown).

### BMP-6 induces upregulation of Id1

**Figure 7:**
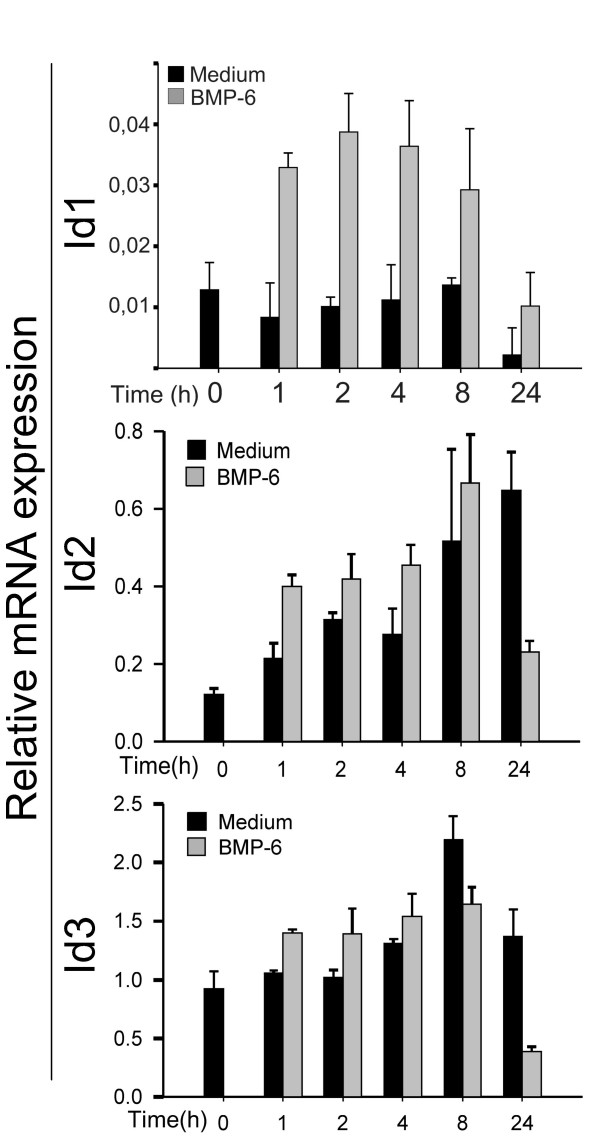
**BMP-6 induced upregulation of Id1 at the mRNA and protein level**. CD19^+ ^B cells were cultured in X-vivo 15 over night before treatment with BMP-6 for the indicated time points. Total RNA was extracted and *Id1*, *Id2 or Id3*-expression was analysed by real-time RT-PCR; values are normalised to the expression level of PGK1 mRNA and expressed as relative quantification (2^-average ^ΔΔC_T _– relative to *Id1*, *Id2 or Id3 *expression in the cell-line Ramos). One representative of three independent experiments is shown.

Next, we wanted to explore whether the BMP-6 induced phosphorylation of Smad 1/5/8 also could induce transcriptional changes of target genes. In this regard, the inhibitors of DNA binding proteins (Ids) are considered to be some of the major target genes for Smad-signalling [17]. Thus, B cells were pre-incubated over night in X-VIVO 15, and then cultured in medium alone or in the presence of BMP-6 for various time points before preparation of total RNA. The amount of *Id1–Id4 *mRNA was quantified by real-time RT-PCR. Interestingly, we observed a specific four-fold upregulation of *Id1 *mRNA in BMP-6-treated B cells (Figure 7). The up-regulation of *Id1 *mRNA was characteristic of an early inducible gene, with maximal upregulation two hours after the addition of BMP-6 and returned to baseline after 24 hours. In contrast, no significant changes were observed for *Id2 *and *Id3 *mRNA, whereas *Id4*-transcripts were not detectable (Figure 7, data not shown).

**Figure 8:**
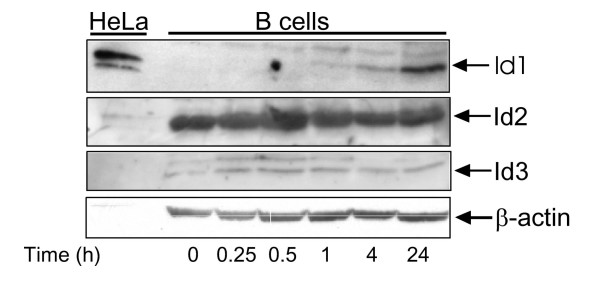
Id protein expression was determined by western-blot analysis. CD19^+ ^B cells were cultured in X-vivo 15 over night before treatment with BMP-6 for the indicated time points and cell lysates were prepared. One representative experiment of three is shown. HeLa cells were used as a positive control for Id1 protein detection.

**Figure 9:**
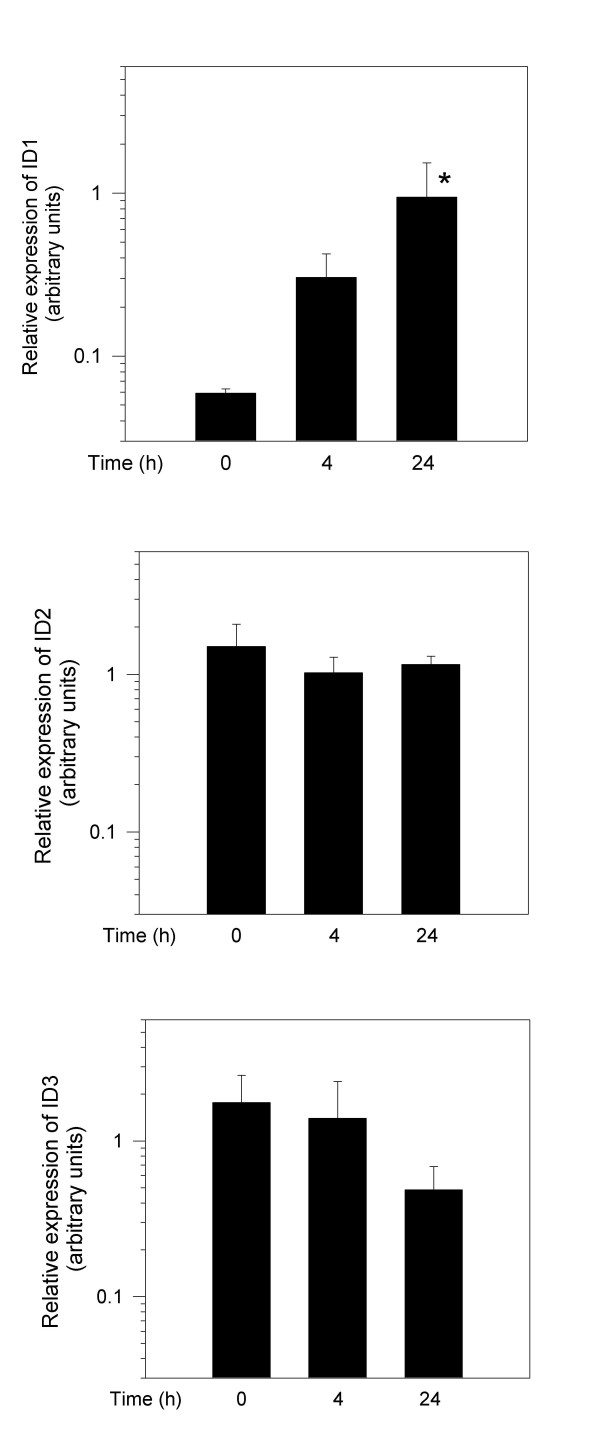
Relative protein expression of Id1, Id2 and Id3. Quantifications of Id1, Id2 and Id3 protein levels were performed using β-actin as normalization and expressed as mean ± SEM (Id1: n = 4, *p ≤ 0.020; Id2 and Id3: n = 3).

Western blot analysis revealed that the BMP-6-induced upregulation of Id1 mRNA also was correlated with upregulation of Id1 protein as well. The increase in Id1 protein level was detectable after one hour and increased until 24 hours after BMP-6 addition, showing a 16-fold upregulation compared with t0 (p ≤ 0.020, n = 4) (Figure 8 and 9). In line with the mRNA data, no consistent change in the amounts of Id2 and Id3 protein could be observed (Figure 8 and 9). We were able to block the Id1 specific band with a blocking peptide (data not shown). Taken together, these data suggest that Id1 could be a possible target gene for mediating the effects of BMP-6 in human B cells, whereas Id2 and Id3 not seem to be involved.

### BMP-6 production in B cells

**Figure 10:**
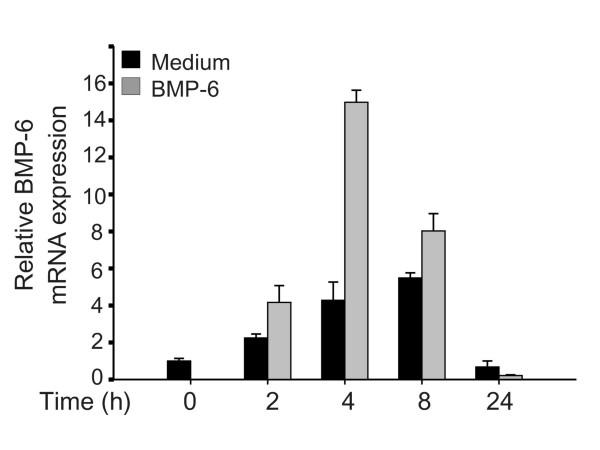
**Anti-IgM rapidly upregulates BMP-6 mRNA expression in human B-cells**. CD19-positive B cells were cultured in X-vivo 15 over night and stimulated with anti-IgM for the indicated time periods, before total RNA was extracted. The expression of *BMP-6 *mRNA by realtime RT-PCR; values are normalised to the expression level of PGK1 mRNA and expressed as relative quantification (2^-average ^ΔΔC_T _– relative to *BMP-6 *expression in the cell-line Ramos. One representative of five independent experiments is shown.

**Figure 11:**
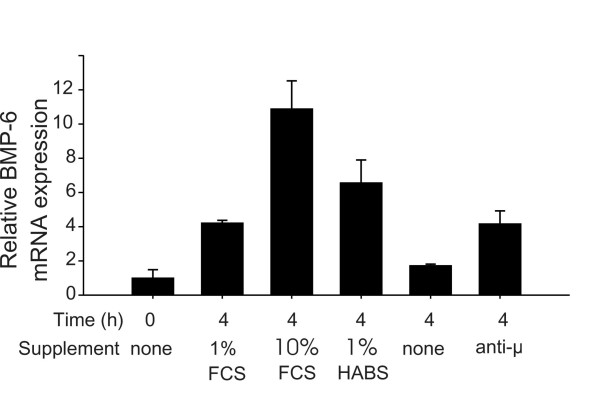
**Fetal calf serum and human AB serum upregulates BMP-6 mRNA expression in human B-cells**. CD19-positive B cells were cultured in X-vivo 15 over night and with fetal calf serum or human AB serum at the indicated dilutions for four hours, before total RNA was extracted. The expression of *BMP-6 *mRNA by realtime RT-PCR; values are normalised to the expression level of PGK1 mRNA and expressed as relative quantification (2^-average ^ΔΔC_T _– relative to *BMP-6 *expression in the cell-line Ramos. One representative of three independent experiments is shown.

The fact that BMP-6 has been reported to act as an autocrine stimulator in chondrocytes [18] and ovarium [19], prompted us to investigate whether normal human B cells could produce *BMP-6 *upon stimulation. Ramos cells, which have been described to express *BMP-6 *mRNA endogenously [20], and the T cell line Jurkat, served as positive and negative controls, respectively. Endogenous *BMP-6 *mRNA levels in normal B cells were quantified by real-time RT-PCR after stimulation with anti-IgM for different time points. Interestingly, the up-regulation of *BMP-6 *mRNA was characteristic of an early-to intermediate inducible gene with maximal upregulation four hours after the addition of anti-IgM. The level of *BMP-6 *mRNA was back to baseline after 24 hours upon stimulation (Figure 10). Furthermore, both FCS and human AB-serum induced significant upregulation of *BMP-6 *mRNA (Figure 11). Interestingly, in a separate study we have found that normal human T cells do not express *BMP-6 *mRNA after activation (Sivertsen et al, manuscript in preparation). Next, we wanted to detect BMP-6 protein in normal B-cells and tested various commercially available antibodies. However, in our hands these anti-BMP-6 antibodies did only recognize the recombinant BMP-6 protein and not the native protein.

## Discussion

Recent studies have demonstrated an important role for BMP superfamily members in hematopoietic stem cells, early thymocytes [6,7] and B-cell malignancies [8,11,12], but a role for BMPs in normal human B cells has previously not been reported. The present study demonstrated a significant antiproliferative effect of BMP-6 in peripheral blood CD19^+ ^B cells. Additionally, BMP-6 induced cell death in CD27^+ ^memory B cells as well as in a Burkitt lymphoma cell line (Ramos). Importantly, BMP-6 induced a rapid and marked increase in Smad-1/5/8 phosphorylation. Furthermore, the BMP-6 induced Smad phosphorylation was followed by a selective upregulation of *Id1 *mRNA and subsequent Id1 protein.

In the present study, the demonstrated antiproliferative effect of BMP-6 in anti-IgM treated B cells was significant and dose-dependent. Importantly, the anti-proliferative effect of BMP-6 could be completely neutralized by the use of a natural inhibitor, Noggin. This is in line with others, showing that Noggin can function as a BMP-6 antagonist [21,22]. In addition, the combination of soluble BMP-RIB-Fc and BMP-RII-Fc fusion proteins also neutralized the anti-proliferative effect of BMP-6 in human B cells. Interestingly, as for other TGF family members, bifunctional effects have also been demonstrated for BMPs. Whereas several of the BMPs have been shown to promote proliferation in various cell types including condrocytes [23], liver [24] and granulosa cells [25], antiproliferative effects and induction of apoptosis has been reported for B and T lineage cells. Similar effects as demonstrated for BMP-6 on human B cells in the present study, were demonstrated for BMP-2, 4, 6 and -7 in human myeloma cells [9-11]. Other members of the BMP-family have also been reported to induce apoptosis, including in mouse B lineage cells [26]. Additionally, BMP-4 inhibits thymocyte proliferation [6]. Taken together, these data suggest that the role of BMPs in the regulation of proliferation and apoptosis is highly cell type dependent.

To examine how BMP-6 exerts its functional effects in B cells, we analysed BMP receptor expression by western blot analysis. Human peripheral B cells were found to express the BMP type I receptors Act-RIA and BMP-RIB, and the type II-receptors BMP-RII and Act-RIIb, which signal after binding of several BMPs, including BMP-6 [16,13]. To further explore BMP-6 induced signalling, activation of several pathways is possible. The major signalling pathway known to date, is activation of R-Smads [13,27]. In that respect, BMPs have been shown to exert antiproliferative effects in B lineage cells via phosphorylation of R-Smad [11,28]. Furthermore, BMP-2 has been shown to induce activation of STAT3 in myeloma cells [9]. However, phosphorylation of R-Smad was not investigated in that study. BMP-2 has also been shown to induce phosphorylation of p38 [29]. Thus, phosphorylation of p38, STAT3 and Smad1/5/8 represent important BMP-signalling pathways that mediated the effects of BMPs and even cross-talk between these pathways has been reported [29,30]. In the present study, we were not able to detect BMP-6-induced changes in the phosphorylation status of STAT3 or p38 in human peripheral B cells. Instead, a rapid and marked phosphorylation of Smad1/5/8 was revealed. In a parallel study, we have found that other BMPs also induced phosphorylation of Smad1/5/8 in peripheral B cells (data not shown). We are currently pursuing microarray studies to identify the signalling pathways and target genes that are differently regulated by the various BMPs in human B cells.

Upregulation of *Id1 *via Smad1/5/8 phosphorylation is a known mechanism for BMP-6 signalling in other cell systems [31,32] and regulation of Id-proteins is thought to be an important mechanism for Smad-signalling [17]. In the present study, real-time RT-PCR experiments revealed a specific four-fold upregulation for *Id1 *in BMP-6-treated B cells, while the amount of *Id2–Id4 *remained unchanged. In agreement with this, western blot analysis demonstrated an upregulation of Id1 protein, while the amount of Id2 and Id3 protein levels remained unchanged. Previously, *Id1 *has been considered not to be expressed in later developmental stages than pro-B cells [33,34], and its constitutive expression has been reported to impair mouse B cell development [35]. Therefore, our demonstration of the time-dependent upregulation of Id1 mRNA and protein in mature normal human B cells is of particular interest. In that respect, it is noteworthy that TGF-β signalling in early and mature B cells induces both Id2 and Id3 expression [36,37], but not Id1 (data not shown). Interestingly, these results show that various members of the TGF-β family regulate Id proteins differently. Id2 and Id3 are considered to be the Id proteins mainly expressed in mature B cells [38]. The present study also found Id2 and Id3 protein in B cells to be more highly expressed than Id1 in resting B cells. However, BMP-6 did not induce significant changes in the protein expression of Id2 and Id3. It is believed that Id proteins block differentiation and promote proliferation in various cell types [39,33]. Id proteins act as dominant-negative inhibitors of E-proteins and Pax5 function by forming dimers with these proteins, making them unable to bind DNA. It has been proposed that the balance among E-proteins, Pax5 and Id proteins might have an important role in activated B cells [38]. In that respect, E-proteins have been implicated in both the promotion and inhibition of cell survival and growth at different points in lymphocyte development [40]. The antiproliferative and death inducing effect of BMP-6 in B cells with concomitant upregulation of Id1 protein is therefore in line with the view that Id proteins are required for the induction of growth arrest and apoptosis in B-lymphocyte progenitors by TGF-β [40].

Furthermore, Id proteins are known as important parts of signalling pathways involved in development, cell cycle and tumorigenesis [32]. It is well established that various members of the Id family are overexpressed in a range of human tumours and generally, *Id1 *appears to be the family member most widely overexpressed in a variety of human malignancies [41], including multiple myeloma [42,32]. Additionally, our findings that BMP-6 activates intracellular signalling pathways in human B cells might be of potential pathophysiological significance in lymphoma and inflammation. High *BMP-6 *mRNA expression in DLBCL has been shown to correlate to unfavourable outcome [12]. In this respect, it is of interest that targeted expression of *Id1 *to B-lymphocytes resulted in aberrant B cell development, massive apoptosis, and subsequent development of B cell lymphomas [35]. Moreover, BMP-6 has been suggested to play a role in rheumatoid arthritis (RA) [43,44] and elevated levels of *Id1 *and *Id3 *have been found in the synovia of RA-patients [45]. Altogether, these results point to an important role for Id proteins in the regulation of normal B cell homeostasis and in diseases, where B cells are involved. It will therefore be important to further elucidate the role of Id-1 in human B cells by selective over expression or inhibition of Id-1 gene expression.

Given the role of BMP-6 in mature human B cells demonstrated here, identification of BMP-6 producing cells in vivo with possibility of interaction with naïve and memory B cells might contribute to the understanding of mature B cell biology. High *BMP-6 *mRNA expression in DLBCL has been detected by gene expression profiling [12]. Furthermore, production of *BMP-6 *transcripts in normal activated B cells was detected in the same study. Of note, an autocrine BMP-6 loop has been reported by others in chondrocytes and in the ovarium [46,19,18]. Therefore, we wanted to explore the possibility for an autocrine BMP-6 loop in human B cells. We analysed the expression *BMP-6 *mRNA in peripheral blood B cells by real-time PCR, and report here the upregulation of endogenous *BMP-6 *transcripts after stimulation with FCS, human AB-serum and, most importantly, anti-IgM. However, our attempts to study BMP-6 protein levels were unsuccessful due to problems with unspecific binding of the anti-BMP-6 antibodies tested, and lack of specific staining in control cells known to express *BMP-6 *mRNA. In contrast, the recombinant protein was readily detected. In that respect, few investigators have detected BMP-6 protein in humans, especially in non-pathogenic tissue. The possibility of BMP-6 production in human B-cells is in line with a recent work that reported the production of BMP-6 in mouse B cells, infiltrating the bone marrow of mice with inflammatory arthritis [43]. In this study, a role for BMPs in the inflammatoric process of arthritis was suggested. The upregulation of the *BMP-6*-transcripts after IgM-crosslinking is of pathophysiologic interest [12]. A loss of TGF-β-responsiveness has been suggested to be a critical contribution to malignant transformation [47,48] and similar oncogenic mechanisms have been postulated for BMPs. Lines of evidence suggest [49] that at early stages of carcinogenesis, BMP-6 is not a tumour promoter, but suppresses benign and malignant tumour outgrowth. These findings are in good agreement with previous findings for other TGF-β family members, including TGF-β1 and BMP-4 [50], indicating that cellular context of the BMP target cell might define the various observed effects. In contrast to the upregulation of *BMP-6 *transcript in B cells, we were not able to detect *BMP-6 *transcripts in human peripheral blood CD4^+ ^or CD8^+ ^T cells (resting or stimulated with anti-CD3 and anti-CD28; data not shown), consistent with the findings in T cell lines [20] and T cells in mice [43]. Other potential BMP-6 sources for mature B cells in vivo might be other cells of the immune system or tissue with contact to the hematopoietic system. One well recognized source for BMP-6 production is the human bone and bone marrow stroma [51,8]. Furthermore, it is noteworthy that human umbilical vein endothelial cells (HUVEC) highly express *BMP-6 *mRNA [52], and vascular endothelium has been reported to produce BMP-6 [53]. These studies might imply a role for BMP-6 in transendothelial migration of B cells. *BMP-6 *mRNA has been demonstrated in murine macrophage cell lines, but not in humans [54]. In accordance with these findings, other human cell lines of neutrophil and monocytic origin have been described to be negative for the *BMP-6 *transcript [20]. To our knowledge, there is currently no report about BMP-6 production of human dendritic cells.

## Conclusion

In conclusion, our results show that BMP-6 induces activation of intracellular Smad signalling in mature human B-cells with consecutive production of Id1 protein. Furthermore, we report that BMP-6 has an antiproliferative effect in B cells stimulated with anti-IgM alone or the combined action of anti-IgM and CD40L. Additionally, BMP-6 induces cell death in activated memory B cells and Ramos cells. Taken together, these results provide a rationale to further examine the role of BMP-6 signalling in normal B cell biology as well as in pathologic conditions like B cell malignancies and autoimmune disorders.

## Methods

### Cell culture

If not specified, all cells were cultured in X-VIVO 15™ (BioWhittaker, Verviers, Belgium) serum-free medium at 37°C and 5% CO_2 _in air.

Peripheral blood was provided by the Blood Bank at Buskerud Regional Hospital with formal agreement by the patients, and approval by the regional ethics committee. Highly purified resting human B-lymphocytes (CD19^+ ^cells) were isolated from the peripheral blood by rosetting with immunomagnetic beads (Dynabeads M450; Dynal, Oslo, Norway) as described [55]. This procedure yields less than 0.5% T cells, 0.1% NK cells, and 0.5% monocytes as judged by indirect immunofluorescence staining.

The following cell lines from human lymphoid malignancies were maintained in RPMI 1640 (PAA Laboratories GmbH, Pasching, Austria) supplemented with 10% foetal bovine serum (FCS), 100 units/ml penicillin G, and 100 units/ml of streptomycin sulphate, but serum-starved for at least four hours and cultured in X-VIVO 15™ when included in experiments: EBV-negative BL cell lines Ramos (ECACC 85030802), HL60 (JCRB0085).

### Growth factors/supplements

The following reagents were used at indicated concentrations: recombinant human (rhu) BMP-6 (1 μg/ml, if not specified otherwise), rhu BMP-RIB/ALK-6/Fc Chimera (5 μg/ml), rhu BMPR-II/Fc Chimera (5 μg/ml), and recombinant mouse Noggin (5 μg/ml) were purchased from R&D Systems (Abingdon, UK); Anti-IgM F(ab)2 fragments of rabbit polyclonal antibodies to human IgM heavy chain (37.5 μg/ml) was obtained from Dako, Copenhagen, Denmark and rhu CD40 ligand (CD40L, 10 ng/ml) was a gift from Immunex Corp. (Seattle, WA).

### Antibodies used for flow cytometric analysis and immunoblot analysis

Antibodies against the human BMP-receptors Act-RIA, BMP-RIB, BMPR-II, Act-RIIA and Act-RIIb were purchased from R & D Systems (Abingdon, UK). Detection of the BMP-6-protein has been tried with the following antibodies: goat polyclonal anti-BMP-6 (Santa Cruz, San Diego, CA, USA), monoclonal mouse anti-BMP-6 and polyclonal goat anti-BMP-6 from R & D Systems (Abingdon, UK), and mouse monoclonal anti-BMP-6 (Chemicon International Inc, Temecula, CA, USA).

Characterisation of BMP-signalling pathways was done by use of anti-phospho-Smad1, -5, -and 8 polyclonal antibody (Chemicon, Temecula, CA, USA). Expression levels of Id1–3 proteins were detected with polyclonal rabbit antibody and detection was blocked with blocking peptide from Santa Cruz Biotechnology (Santa Cruz, San Diego, CA, USA). As secondary antibodies served anti-mouse, anti-goat or anti-rabbit IgG- horseradish peroxidase (HRP) from Dakocytomation AS (Copenhagen, Denmark) for immunoblot analysis. Anti-β-actin was from Santa-Cruz. From Becton Dickinson (San Jose, CA), we purchased anti-CD19-PE, anti-CD19-FITC. The antibodies used for cell sorting were anti-CD19 PC5 from Immunotech SA (Marseille, France) and anti-CD27 PE from Becton Dickinson, Biosciences Pharmingen (San Diego, CA, USA).

### Cell sorting

Highly purified CD19^+^CD27^- ^or CD19^+^CD27^+ ^cells were obtained by staining CD19^+ ^cells with anti-CD27 PE and CD19 PC5 mAbs for 30 minutes at 4°C, followed by washing with PBS and sorting on FACS DiVa from Becton Dickinson.

### Western-blot analysis

B cells from peripheral blood or cultured cell-lines were lysed in lysis buffer (glycerol 10%, β-mercaptoethanol 5%, 0.0625 M Tris-HCL [pH 6.8], sodium dodecyl sulphate [SDS] 2.5%w/vol). Total protein (30–100 μg) from each sample was run on 10% or 12% SDS/polyacrylamide (SDS/PAGE) gels and blotted onto nitrocellulose filters (Protran; Schleicher &Schuell GmbH, Dassel, Germany). Blocking, washing and incubation of the filters with primary antibodies were done according to the manufacturer's protocols at room temperature (RT). After washing with TBS/0.1% Tween-20 (TBS-T), the filters were incubated with horseradish peroxidase (HRP) coupled to relevant secondary antibodies (see above) for 60 minutes at RT. Enzyme activity was visualised by the enhanced chemiluminescence system, ECL^+^PLUS (Amersham, Buckinghamshire, UK). Densitometric analysis was performed by scanning hyperfilms on a Personal Densitometer SI (Molecular Dynamics, Sunnyvale, CA). Quantification of Id1, Id2 and Id3 protein was calculated by normalizing the specific protein bands to β-actin using Image Quant 5.5 software (Molecular Dynamics).

### Analysis of *BMP-6 *messenger RNA (mRNA) expression

Endogenous expression of the BMP-6 gene was examined by reverse transcription-polymerase chain reaction. Total RNA was isolated using Absolutely RNA™ RT-PCR Miniprep Kit (Stratagene Europe, Amsterdam, Netherland) according to the manufacturers instructions. Quantification of the isolated total RNA was achieved by using spectrophotometric OD_260 _measurements. Equal amounts of RNA were then reverse transcribed to cDNA with TaqMan^® ^Reverse Transcription Reagents (Applied Biosystems, Foster City, CA, U.S.A). To measure mRNA expression of *BMP6*, *Id1–Id4 *and PGK1 PCR were carried out with TaqMan^® ^universal master mix. Primers and probes were provided by Assay-on-Demand (Applied Biosystems). PCR reactions were carried out in a final volume of 25 μl (BMP-6) or 20 μl (ID1). The cDNA added to each reaction was equivalent to the input of 20 ng of total RNA. The gene expression was quantified using the standard curve method (*BMP6*), or the comparative C_T _method (Id1) as described in ABI7700 User Bulletin 2 (Applied Biosystems). The expression was then normalized to the expression level of PGK1. PGK1 was chosen, because it has been shown to have low expression variability among lymphocyte specimens [56]. Expression levels in B cells were then related to the expression levels in Ramos cells.

### Cell proliferation

For estimation of DNA synthesis, CD19^+ ^cells (7.5 × 10^4 ^cells/0.2 ml) or Ramos cells (1 × 10^4 ^cells/0.2 ml) were cultured in triplicate in microtiter wells. The cells were pulsed with 3.7 × 10^4^Bq [^3^H]thymidine (Amersham, Buckinghamshire, UK) for the last 16 h of a 72-h incubation. The cells were harvested using an automated cell harvester (Packard Instrument Company, Meriden, CT, USA) and [^3^H]thymidine incorporation was determined in a scintillation counter (TopCount, Packard Instrument Company Inc., Meriden, CT).

### Determination of cell death

Cell death was measured by vital dye exclusion test by staining cells with 5 μg/ml propidium iodide ([PI]; Calbiochem Corp.; La Jolla, CA; 5 mg/ml) for one minute on ice. At least 1,000 cells per sample were run on a BD FACSCalibur flow cytometer.

### Statistical analysis

The statistical significance of differences between groups was determined using the paired two-tailed Wilcoxon nonparametric test, by applying SPSS10.1 software (SPSS Inc., Chicago, IL, USA). *P *values less than 0.05 were considered significant.

## List of abbreviations

BMP, Bone morphogenetic protein

DLBCL, diffuse large B cell lymphoma

Id, inhibitor of dna binding

Smad, the name Smad originates from the *Drosophila *protein, MAD and the *Caenorhabditis elegans *proteins, sma-2, 3 and 4

STAT, signal transducer and activator of transcription

## Authors' contributions

CK designed and conducted experiments, oversaw research, and wrote paper. EAS designed and conducted experiments, oversaw research. MEH designed and conducted experiments, oversaw research. LF designed and conducted experiments. EBS designed experiments, oversaw research, and wrote paper. JHM designed and conducted experiments, oversaw research, and wrote paper.
